# Impact of optic disc hemorrhage on subsequent glaucoma progression in mild-to-moderate myopia

**DOI:** 10.1371/journal.pone.0189706

**Published:** 2017-12-18

**Authors:** Ahnul Ha, Young Kook Kim, Jin Wook Jeoung, Ki Ho Park

**Affiliations:** 1 Department of Ophthalmology, Seoul National University College of Medicine, Seoul, Korea; 2 Department of Ophthalmology, Seoul National University Hospital, Seoul, Korea; Massachusetts Eye & Ear Infirmary, Harvard Medical School, UNITED STATES

## Abstract

**Purpose:**

To investigate optic disc hemorrhage (DH)’s clinical implications to subsequent progression of primary open-angle glaucoma (POAG) in cases of mild-to-moderate myopia.

**Design:**

Retrospective comparative study.

**Participants:**

(1) Fifty-nine (59) myopic (26.5 mm > axial length [AXL] ≥ 24.0 mm) POAG patients with DH and (2) 59 age-, AXL-, and mean deviation (MD) of visual field (VF)-matched controls without DH were evaluated over the course of a minimum 3.5 years of follow up. For clear assessment of the effect of DH on progression of glaucoma, the patients selected for inclusion in the study were those with stable IOP (i.e., those showing an at least 20% reduction relative to the baseline IOP) whose IOP-lowering medication was not increased, supplemented or changed during the follow-up period.

**Methods:**

The patients’ optic disc/retinal nerve fiber layer (RNFL) photographs were independently evaluated by three glaucoma specialists for structural progression of glaucoma. Event-based analysis with Guided Progression Analysis (GPA) software was used to determine their functional progression. The durations of structural and functional progression were compared by Kaplan-Meier life survival analyses.

**Main outcome measures:**

Optic disc/RNFL progression and VF progression.

**Results:**

The mean follow-up periods between the DH and non-DH groups were not significantly different: in the DH group, 5.6±2.7 years; in the non-DH group, 5.4±2.6 years (P = 0.588). In the DH group, 30 (50.8%) of 59 eyes manifested optic disc/RNFL deterioration; in the non-DH group, however, only 17 (28.8%) of 59 eyes showed structural progression. For the DH group, the cumulative probability of structural glaucoma progression was significantly greater than for the non-DH group (P = 0.001; log rank test). Interestingly, the two groups did not significantly differ in the cumulative probability of functional progression (P = 0.79; log rank test): in the DH group, VF progression was observed in 14 eyes (23.7%); in the non-DH group, in 12 eyes (20.3%).

**Conclusions:**

DH was associated with a greater probability of structural progression in medically well-controlled-IOP POAG eyes with mild-to-moderate myopia. However, the relevance of DH to VF progression was not clear over the course of the average 5.5-year duration of the study.

## Introduction

Optic disc hemorrhage (DH) is one of the most important risk factors with respect to glaucoma progression [1–4]. There have been many reports of strong associations between structural glaucoma deterioration and DH. Changes to the optic disc, new retinal nerve fiber layer (RNFL) defects and RNFL-defect enlargement all can occur subsequently to DH [5–7]. A recent relevant report indicated a faster spectral-domain optical coherence tomography (OCT)-measured rate of RNFL thinning after DH than before in the DH quadrant, whereas in the non-DH quadrant, the rates were not significantly different [4]. Also, multiple studies have shown that forms of functional aggravation, for example a faster rate of visual field (VF) change or new glaucomatous VF defect, can be observed after DH [8,9].

The pathophysiology of DH has not yet been fully elucidated, despite its strong association with glaucoma progression. According to the mechanical hypothesis, DH is the consequence of microvascular disruption resulting from structural alteration of the lamina cribrosa (LC) [5,10]; according to the vascular hypothesis, optic DH is associated with compromised blood supply inside the optic nerve head [11,12]. These are in fact the two most compelling theories on DH pathogenesis; indeed they appear to be at least partly related.

In myopic eyes, axial elongation of the eyeball is accompanied by structural changes to the optic disc and/or peripapillary retina [13,14]. Tilted, rotated and/or larger discs are more likely to be seen in myopic individuals [15]. Those with myopia also more frequently show thinning and stretching of the LC and elongation of the optic nerve head’s peripapillary region [16,17]. In this unique myopic microenvironment, the development of DH might be affected by structural vulnerability around optic nerve head. It would be expected, therefore, that myopic glaucoma patients’ clinical-DH implications are not the same as those of non-myopic individuals.

No previous research has clearly demonstrated the effect of DH on subsequent progression in glaucoma eyes with myopia. Thus, we carried out the present study to evaluate myopic patients’ clinical-DH implications for glaucoma progression, specifically by comparing myopic primary open-angle glaucoma (POAG) eyes with and without DH.

## Methods

This study was approved by the Seoul National University Hospital Institutional Review Board and adhered to the tenets of the Declaration of Helsinki.

### Study subjects

Patients who had been examined at Seoul National University Hospital’s Glaucoma Clinic from January 2005 to May 2013 and followed up on at 6-month intervals for a minimum of 3.5 years were consecutively included in this study after a retrospective medical-record review. All underwent a complete ophthalmic examination: visual acuity assessment, refraction, slit-lamp biomicroscopy, gonioscopy, Goldmann applanation tonometry (Haag-Streit, Koniz, Switzerland), and dilated-funduscopic examination. Additionally, they were subjected to the following: central corneal thickness measurement (Orbscan 73 II, Bausch & Lomb Surgical, Rochester, NY, USA), AXL measurement (Axis II PR; Quantel Medical, Inc., Bozeman, MT, USA), digital color stereo disc photography (SDP), red-free RNFL photography, optic nerve head imaging by Cirrus spectral domain-optic coherence tomography SD-OCT; Carl Zeiss Meditec, Dublin, CA), and Humphrey Visual Field central 30–2 threshold tests (HFA II; Humphrey Instruments Inc., Dublin, CA, USA).

Upon enrollment in the study the eligible participants had POAG, mild-to-moderate myopia (AXL between 24.0 mm or longer and less than 26.5 mm) [18,19], and best-corrected visual acuity of 20/40 or better. Patients were diagnosed with POAG based on the following criteria: (1) typical glaucomatous optic neuropathy with corresponding VF loss; (2) normal anterior chamber angles. Glaucomatous optic neuropathy was determined, without regard to the untreated intraocular pressure (IOP) level, based on certain characteristic optic disc and/or RNFL changes (e.g., the presence of diffuse or localized rim thinning, rim notching, and an RNFL defect) as visible on SDP images or red-free RNFL photographs: Diagnosis of glaucomatous VF defect was made based on the following criteria: a “glaucoma hemifield outside normal limits” results for two or more consecutive VF tests or two or more consecutive VF test results indicating at least three contiguous P < 0.01 test points (one or more of which was < 0.05) for the same hemifield on a pattern deviation plot. Note that these tests required, for their validity, fixation loss rate ≤ 20%; false-positive and false-negative error rates ≤ 25%.

Patients were excluded from further analysis any of the following reasons: history of intraocular surgery (exception: uncomplicated cataract); any posterior-pole lesions possibly affecting VF examination results; current usage of oral antiplatelet and/or anticoagulation medication; associated systemic disease(s) other than hypertension and well-controlled diabetes mellitus. All of the included patients’ eyes had well-controlled IOP: that is, all having received one or more topical glaucoma medications, and all showed at least 20% IOP decreases from the baseline. For accurate assessment of DH’s effect on glaucoma progression, only stable-IOP patients for whom IOP-lowering medication was not increased, supplemented or changed until a definite VF progression was found were included in this study. As for those without clinically detected DH, age-, AXL- and MD-of-VF-matched eyes were selected for inclusion in the non-DH group.

### Determination of optic disc area and parapapillary atrophy area

The optic disc area was calculated in consideration of the magnification factors related to the SD-OCT camera and the eye by substituting the subject's AXL as previous study [20]. The *β-*zone parapapillary atrophy (PPA) area (an inner crescent of chorioretinal atrophy with visible sclera and choroidal vessels) and the clinical disc margin were plotted by using a mouse-driven cursor to trace the disc and PPA margins directly onto the SDP image. Then the pixel areas of the *β*-zone PPA and clinical disc were obtained with the ImageJ 1.40 software (http://rsb.info.nih.gov/ij/index.html; provided in the public domain by the National Institutes of Health, Bethesda, MD, USA). The *β* -zone PPA area was calculated from the ratio of PPA pixel area to the disc pixel area as previously described method [21].

### Discrimination of disc hemorrhage

During the follow up, all of the patients had their SDPs repeated every 6 months at the minimum. These images were retrospectively and independently evaluated for DH by three glaucoma specialists (ANH, YKK, and KHP) blinded to the patients’ clinical information. For any of the following reasons, DH was considered to be unrelated to glaucoma: (1) the disc was swollen, or otherwise obviously abnormal, due to non-glaucomatous optic neuropathy; (2) there were multiple proximitous retinal hemorrhages suggestive of either diabetic retinopathy or retinal vascular abnormality; (3) there was acute posterior vitreous detachment possibly causative of DH. In bilateral DH cases meeting all of the inclusion criteria, just one eye was selected randomly. Only patients who had developed DH during interval testing were enrolled in the study.

### Assessment of structural progression

Three experienced graders (ANH, YKK, and KHP) blind to the patients’ clinical and VF information reviewed each patient’s SDPs and RNFL photographs. They then determined, based on the initial photograph, structural progression’s presence or absence. Optic disc progression was considered to be the extent of neuroretinal rim thinning. RNFL progression was defined as widening, deepening, or a newly apparent RNFL defect. If eyes had diffuse atrophy or invisible RNFL, and if evaluation of RNFL photography, consequently, was difficult, progression was determined solely based on optic disc evaluation. In cases of eyes with severe optic disc tilt and/or torsion rendering neuroretinal rim evaluation unreliable, confirmation of progression was made based only on RNFL photography assessment. Each glaucomatous eye was classified by each of the graders as either stable or progressing.

If the opinions of the three examiners on DH discrimination or structural progression differed, post-discussion consensus was reached. If no consensus could be reached, the pertinent eye was excluded from subsequent analysis.

### Assessment of functional progression

The patients’ VF test results were evaluated to assess functional glaucoma progression. The first 1 to 2 VF results were excluded to minimize the learning effects, and any unreliable results (fixation loss rate > 20%, false-positive and false-negative error rates > 25%) were also excluded. VF progression was determined by event-based analysis using the Humphrey Field Analyzer with Guided Progression Analysis (GPA) software. VF progression was defined as a significant decrease from the baseline pattern deviation at three or more of the same test points on two or three consecutive VF tests. The GPA software classifies VF progression as either ‘‘possible progression” or ‘‘likely progression.” In the current study, only ‘‘likely progression” was considered to be VF progression. A glaucoma specialist (YKK) reviewed all the patients’ VF results to ensure that there was no artefactual software analysis included.

### Statistical analysis

The DH and non-DH groups’ demographic and clinical characteristics were compared. The comparison of normally distributed data was performed using the independent Student’s *t*-test. The categorical data, meanwhile, were analyzed by χ^2^ test. The cumulative risk ratios of structural and functional progression were compared between the groups by Kaplan-Meier survival analysis and log rank test. The initial progression detection was regarded as the endpoint in the survival analysis. The end of follow up was deemed to be the point at which patients without progression were censored. The statistical analysis was performed using the SPSS statistical package (SPSS 22.0; Chicago, IL, USA). A *P* value less than 0.05 was considered to be statistically significant.

## Results

A total of 1048 myopic glaucoma patients’ medical records were initially reviewed for the purposes of the present study, and 80 DH eyes that met the inclusion and exclusion criteria were selected. One (1) case was excluded from subsequent analysis because the opinions of the three examiners on structural progression differed, and no consensus could be reached. Among the remaining subjects, 3 eyes were excluded from further analysis due to myopia-related retinopathy diagnosed over the course of follow-up. Seventeen (17) patients who had received post-DH treatment reinforcement immediately after the occurrence of DH also were excluded.

The DH group finally included 59 myopic glaucoma patients. The mean follow-up period after initial DH was 5.6 ± 2.7 years. 13 eyes (22.0%) of DH group showed multiple DHs during the follow-up periods. 8 eyes had 2 episodes of DH, 5 eyes had more than 2 episodes of DH. For the purposes of comparative analysis, age-, AXL- and baseline MD-of-VF-matched controls (59 patients) who did not incur DH during the follow-up period were included in the non-DH group. Their mean follow-up period (5.4 ± 2.6 years) did not significantly differ from those of the DH group (Table 1).

**Table 1:** Clinical characteristics of myopic primary open angle glaucoma patients with and without optic disc hemorrhage.

	DH group (n = 59)	Non-DH group (n = 59)	*P*
Age (yrs)	55.9 ± 12.4	55.5 ± 12.3	0.859^†^
Gender (male / female)	34 / 25	35 / 24	0.852^‡^
DM (positive / negative)	6 / 53	7 / 52	0.769^‡^
HTN (positive / negative)	14 / 45	17 / 42	0.530^‡^
Untreated baseline IOP (mmHg)	16.6 ± 4.1	16.2 ± 4.5	0.508^†^
Initial IOP** (mmHg)	13.5 ± 2.3	13.3 ± 2.5	0.669^†^
IOP reduction at final follow-up (%)	22.4 ± 14.6	22.2 ± 16.2	0.928^†^
Mean IOP (mmHg)	13.8 ± 1.6	14.0 ± 1.3	0.781^†^
IOP fluctuation (mmHg)	1.4 ± 0.6	1.3 ± 0.7	0.933^†^
CCT (㎛)	537 ± 36.1	531 ± 34.2	0.402^†^
Spherical equivalent (D)	-5.03 ± 3.3	-4.53 ± 3.6	0.447^†^
Axial length (mm)	25.7 ± 1.1	25.8 ± 1.2	0.706^†^
Optic disc area (mm^2^)	2.2 ± 0.1	1.9 ± 0.3	0.100^†^
PPA area (mm^2^)	1.1 ± 0.7	1.0 ± 0.6	0.761^†^
Initial VF MD (decibels)	-4.69 ± 5.2	-4.54 ± 5.0	0.876^†^
Initial VF PSD (decibels)	6.7 ± 5.1	6.1 ± 4.9	0.491^†^
Number of SDP/RNFL photography	11.2 ± 3.58	11.0 ± 3.49	0.809^†^
Number of VF examinations	8.1 ± 1.9	8.0 ± 1.9	0.810^†^
Total follow-up period (mos)	67.6 ± 31.9*	64.4 ± 31.1	0.588^†^
Mean follow-up interval (mos)	6.2 ± 0.3	6.1 ± 0.2	1.000^†^

Values are mean ± standard deviation.

DM, diabetes mellitus; HTN, hypertension; D, diopters; IOP, intraocular pressure; CCT, central corneal thickness; PPA, parapapillary atrophy; VF, visual field; MD, mean deviation; PSD, pattern standard deviation; SDP, stereo disc photography; RNFL, retinal nerve fiber layer

* Defined as the period after the date of initial disc hemorrhage.

** Defined as the IOP at the time of study enroll.

† Student t test

‡ Chi-square test.

Table 1 summarizes the patients’ demographic characteristics. In the DH group, the baseline mean age was 55.9 ± 12.4 (30–88) years. The spherical equivalent of refractive error ranged from -7.13 to -0.50 D (-5.03 ± 3.3 D); AXL, from 24.04 to 26.45 mm (25.7 ± 1.1 mm); treated IOP, from 8.3 to 16.7 mmHg (13.8 ± 1.6 mmHg); initial VF MD, from -11.4 to +0.12 dB (-4.69 ± 5.2 dB). In the non-DH group, on the other hand, the baseline mean age was 55.5 ± 12.3 (30–88) years. The spherical equivalent of refractive error ranged from -7.50 to -0.25D (-4.53 ± 3.6 D); AXL, from 24.03 to 26.35 mm (25.8 ± 1.2 mm); treated IOP, from 8.5 to 16.4 mmHg (14.0 ± 1.3 mmHg); initial VF MD, from -10.8 to -0.02 dB (-4.54 ± 5.0 dB). No significant differences were found between the two groups with regard to the above characteristics. Also, none of the following basic demographics and clinical characteristics significantly differed between the groups: systemic comorbidities, rate of IOP reduction from baseline, amount of IOP fluctuation, central corneal thickness and PPA area.

In the DH group (59 eyes), progression was observed in 30 eyes (50.8%) by optic disc/RNFL photographic assessment, in 14 eyes (23.7%) by VF analysis, and in 38 eyes (64.4%) by either optic disc/RNFL or VF examination. The mean intervals between DH and structural/VF progression were 2.7 ± 1.1 and 6.1 ± 2.2 years, respectively.

In the non-DH group, 17 eyes (28.8%) showed progression by optic disc/RNFL photographic assessment, 12 eyes (20.3%) by VF analysis, and 19 eyes (32.2%) by either optic disc/RNFL or VF exams. The mean intervals between the baseline and structural/VF progression were 3.5 ± 2.33 and 5.6 ± 2.45 years, respectively.

**Fig 1:**
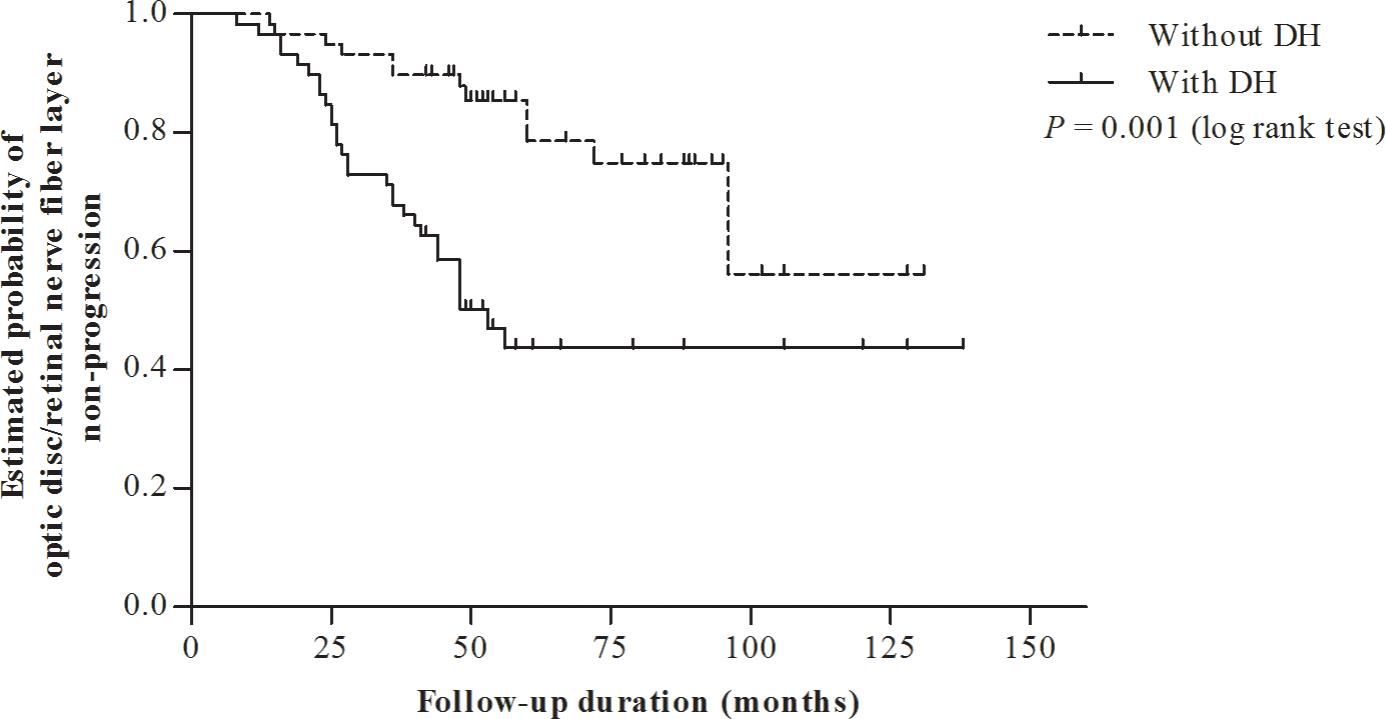
Kaplan–Meier analysis of estimated probability of optic disc/retinal nerve fiber layer (RNFL) non-progression in patients with disc hemorrhage (DH) versus those without DH. The cumulative probability of optic disc/RNFL non-progression (10-year survival rate: 0.44 ± 0.01) was significantly lower in the DH group than in the non-DH group (10-year survival rate: 0.47 ± 0.12) (P = 0.001, log rank test).

**Fig 2:**
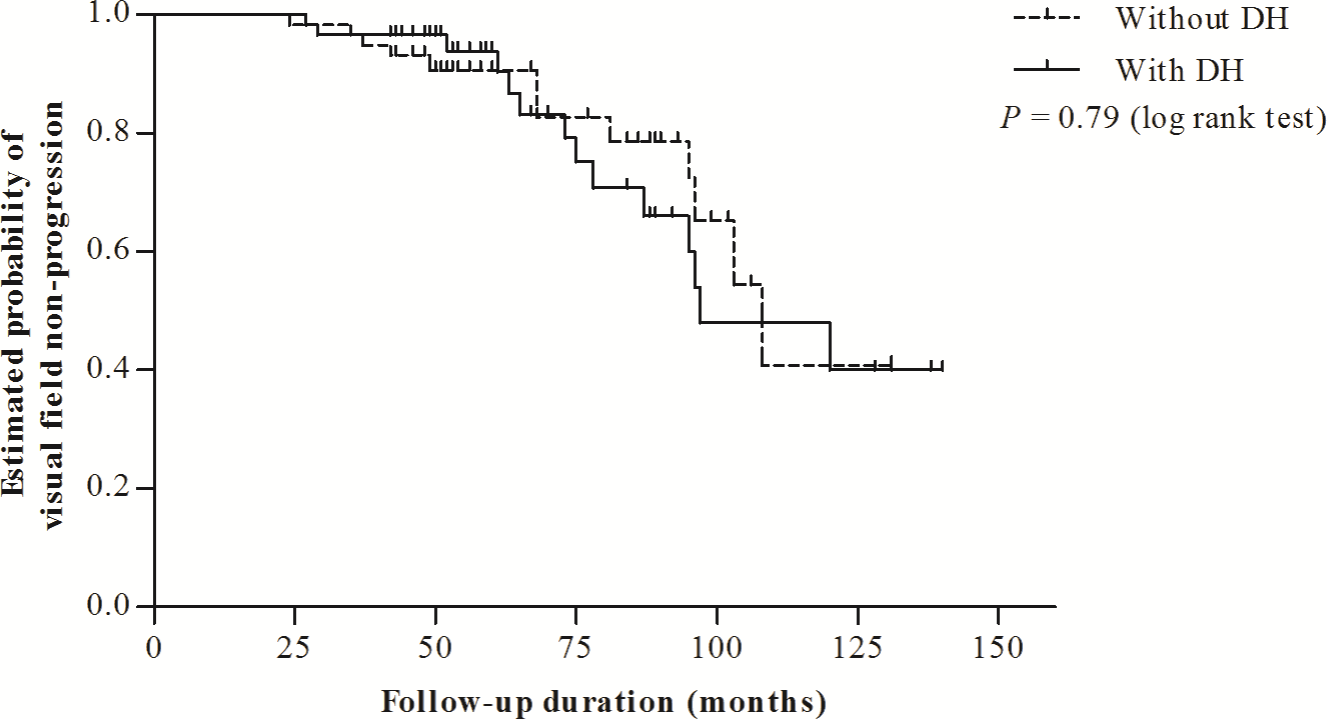
Kaplan–Meier analysis of probability of maintained non-deterioration of visual field (VF) in patients with disc hemorrhage (DH) and in those without DH. The cumulative probability of non-progression of VF (10-year survival rate: 0.40 ± 0.12) was statistically equivalent between the DH and non-DH groups (10-year survival rate: 0.41 ± 0.15) (P = 0.79, log rank test).

The cumulative probability of structural progression was significantly greater for the DH group than that for the non-DH group (P = 0.001, log rank test, Fig 1). However, Kaplan-Meier survival analysis showed that in fact, the patients with and without DH shared a cumulative probability of VF non-progression that was statistically equivalent (P = 0.79, log rank test, Fig 2). The detailed data used in Kaplan-Meier survival analysis is shown in S1 Table.

**Fig 3:**
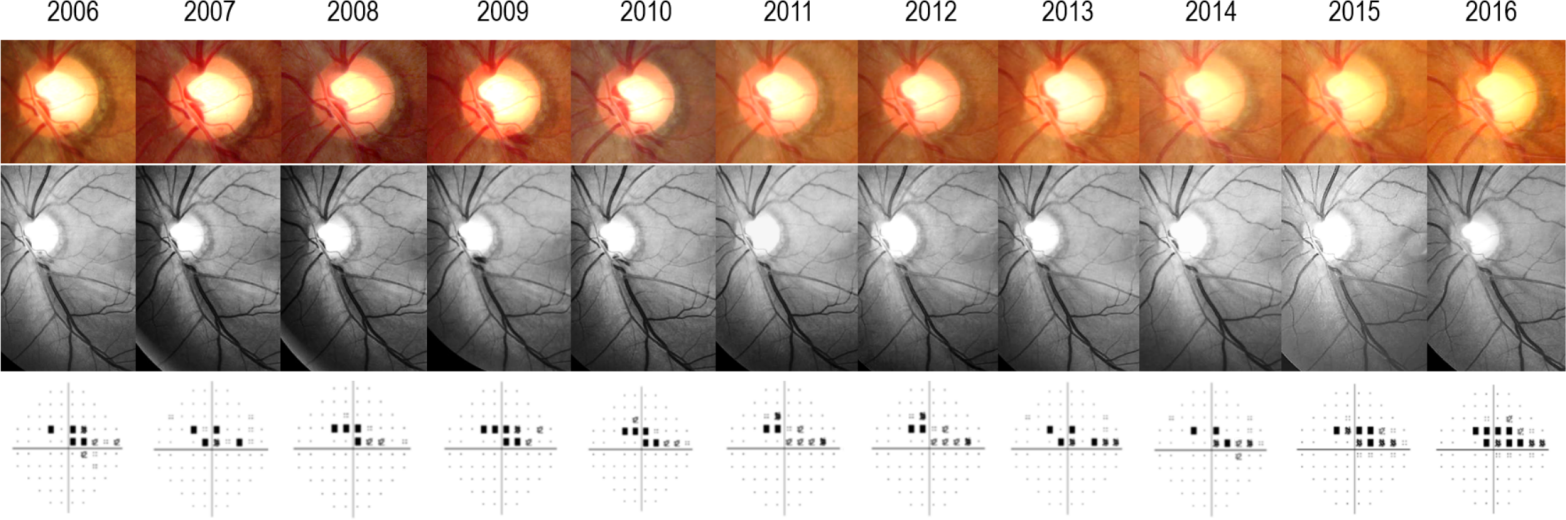
Representative case of disc hemorrhage (DH) in primary open-angle glaucoma (POAG) with mild-to-moderate myopia. The first row contains stereo disc photographs (SDPs) showing recurrent DH in inferotemporal optic disc region (between 2006 and 2010) and subsequent neuroretinal rim thinning. The second row contains red-free retinal nerve fiber layer (RNFL) photography representing gradual enlargement of RNFL defect 3.8 years after initial clinical detection of DH (in 2010). The last row shows visual field progression manifested 9.0 years following the initial DH (in 2015).

Fig 3 provides a representative case (male, age 40 at time of DH; AXL 25.25 mm; SE -4.125) in which optic disc/RNFL photographic progression was clear at post-DH 3.8 years but where VF progression manifested 9.0 years following the initial DH.

## Discussion

The current study demonstrated a higher cumulative progressive-optic-disc-deterioration (including RNFL progression) probability for myopic POAG eyes with DH than for eyes without DH: of the patients who showed post-DH structural progression in this study, 88% of the eyes manifested DH-adjacent RNFL defect progression. The association between DH and progressive glaucomatous changes already are well documented [2–4]. Nitta et al. suggested that in normal-tension glaucoma eyes, RNFL-degenerative changes and damage to the capillary network that surrounds the RNFL-defect border can induce DH [5]. Quigley et al. claimed that rapid rim-tissue degeneration results in microvasculature stress, thereby causing DH [10]. With the advancement of OCT, the association between LC-structural alteration and DH has been elucidated [22–25]. Based on the results of the current study, we can deduce that DH might represent a vulnerable site for damage to optic disc and/or RNFL, even in myopic eyes.

Interestingly, we were unable to show statistically significant differences between our two groups (DH and non-DH) in VF progression. That is, the relevance of DH to VF progression was not clear over the course of the average 5.5-year duration of the study. In glaucoma, it has been well known that structural damage precedes functional deterioration [26–28]. Previous studies on glaucomatous eyes have reported that RNFL defect might precede glaucomatous VF-loss onset by as much as 6 years [27,29]. The current study’s mean follow-up period, 5.5 years, might not be long enough to reveal functional deterioration resulting from structural changes in myopic eyes. Additionally, more frequent VF testing has been suggested as a higher-sensitivity means of detecting functional progression [30]. Chauhan et al. concluded that a sufficient number of reliable VFs in the early follow-up period is essential to more accurate assessment of VF progression [31]. If VF examinations had been performed more frequently in the current study, we might have found a difference in the probability of VF progression between the two groups (DH and non-DH). Furthermore, our study population was comprised of early-glaucoma patients for whom the baseline MD of VF averaged -4.69 dB and -4.54 dB in the DH and non-DH groups, respectively. The greater progression-detection sensitivity of serial RNFL photography relative to VF examination during the early-glaucoma stage might have influenced the current study’s structural and functional progression results [32].

For clear assessment of the effect of DH on progression of glaucoma, the patients selected for inclusion in the study were those with stable IOP (i.e., those showing an at least 20% reduction relative to the baseline IOP) [33,34] whose IOP-lowering medication was not increased, supplemented or changed during the follow-up period. Debate still rages over the need for post-DH treatment modification in clinical care settings. Medeiros et al. compared glaucoma patients’ mean VF progression rates before and after DH, their data showing that greater IOP reduction resulting from more aggressive treatments after DH contributed to a slower rate of MD decrease [35]. Recently, Tadamichi et al. identified the benefits of treatment reinforcement for reduced rate of post-DH RNFL thinning [4]. However, considering the relatively slow rate of post-DH functional progression revealed in the present study, further, longer-follow-up investigations should be conducted to determine if post-DH treatment enhancement can be beneficial to slowing down progression in myopic POAG patients with stable IOP.

The present study’s findings should be interpreted with mindfulness of its limitations. First, we cannot exclude the possibility of selection artifacts. It is possible that patients whose DH was missed were included in the non-DH group, because whereas DH has been reported to last for only 2 to 6 months, disc photographs were recorded at 6-month intervals. However, misclassification of patients with DH to non-DH group would bias the results toward the null. So statistically significant difference that found between the groups in this study might imply even stronger association. Certainly, the frequency of follow-up would impact DH- and progression-detection rates. Although the average numbers of SDP/RNFL photographs and VF examinations were equivalent in the groups with and without DH, there is a possibility that patients at greater risk had been followed up more frequently. Second, glaucomatous progression for each patient was evaluated only after the development of DH, and deterioration before the event was not assessed. Such analysis would require that all patients had at least 5 VF tests before DH detection and sufficient follow up duration, which was not available for most of the enrolled subjects. De Moraes et al. reported that spatially consistent VF progression may occur before DH detection [9]. Third, we studied a group of mostly normal-baseline-IOP POAG eyes (95.8% of the subjects had a baseline IOP equal to or less than 21 mmHg); thus, our results might not be directly applicable to other OAG populations. Fourth, the relatively small number of study patients and insufficient follow-up period might have affected the study results. Further research with larger patient cohorts and longer follow-up durations would be expected to confirm whether VF-defect-progression differences between DH and non-DH groups exist or not in cases of myopic POAG.

In conclusion, DH was associated with a greater probability of optic nerve head/RNFL progression in POAG eyes with mild-to-moderate myopia. Over the course of the 5.5-year follow up, however, there were no significant differences in VF-deterioration probability between myopic POAG eyes with and without DH. Determination of whether post-DH treatment reinforcement can be of any significant benefit to myopic POAG eyes with stable IOP will have to await further study.

## Supporting information

S1 Table Structural and functional progression data in patients with disc hemorrhage (DH) and those without DH. (XLSX)
