# Correction: Impact of optic disc hemorrhage on subsequent glaucoma progression in mild-to-moderate myopia

**DOI:** 10.1371/journal.pone.0198647

**Published:** 2018-05-31

**Authors:** Ahnul Ha, Young Kook Kim, Jin Wook Jeoung, Ki Ho Park

## Notice of republication

This article was republished on May 21, 2018 to replace an incorrect version of S1 Table. Please download this article again to view the corrected table.
